# Packaging Health Services When Resources Are Limited: The Example of a Cervical Cancer Screening Visit

**DOI:** 10.1371/journal.pmed.0030434

**Published:** 2006-11-14

**Authors:** Jane J Kim, Joshua A Salomon, Milton C Weinstein, Sue J Goldie

**Affiliations:** 1 Department of Health Policy and Management, Harvard School of Public Health, Boston, Massachusetts, United States of America; 2 Department of Population and International Health, Harvard School of Public Health, Boston, Massachusetts, United States of America; Institutionen for Medicinsk Epidemiologi och Biostatistik, Sweden

## Abstract

**Background:**

Increasing evidence supporting the value of screening women for cervical cancer once in their lifetime, coupled with mounting interest in scaling up successful screening demonstration projects, present challenges to public health decision makers seeking to take full advantage of the single-visit opportunity to provide additional services. We present an analytic framework for packaging multiple interventions during a single point of contact, explicitly taking into account a budget and scarce human resources, constraints acknowledged as significant obstacles for provision of health services in poor countries.

**Methods and Findings:**

We developed a binary integer programming (IP) model capable of identifying an optimal package of health services to be provided during a single visit for a particular target population. Inputs to the IP model are derived using state-transition models, which compute lifetime costs and health benefits associated with each intervention. In a simplified example of a single lifetime cervical cancer screening visit, we identified packages of interventions among six diseases that maximized disability-adjusted life years (DALYs) averted subject to budget and human resource constraints in four resource-poor regions. Data were obtained from regional reports and surveys from the World Health Organization, international databases, the published literature, and expert opinion. With only a budget constraint, interventions for depression and iron deficiency anemia were packaged with cervical cancer screening, while the more costly breast cancer and cardiovascular disease interventions were not. Including personnel constraints resulted in shifting of interventions included in the package, not only across diseases but also between low- and high-intensity intervention options within diseases.

**Conclusions:**

The results of our example suggest several key themes: Packaging other interventions during a one-time visit has the potential to increase health gains; the shortage of personnel represents a real-world constraint that can impact the optimal package of services; and the shortage of different types of personnel may influence the contents of the package of services. Our methods provide a general framework to enhance a decision maker's ability to simultaneously consider costs, benefits, and important nonmonetary constraints. We encourage analysts working on real-world problems to shift from considering costs and benefits of interventions for a single disease to exploring what synergies might be achievable by thinking across disease burdens.

## Abstract 2

Jane Kim and colleagues analyzed the possible ways that multiple health interventions might be packaged together during a single visit, taking into account scarce financial and human resources.

## Editors' Summary

**Background.:**

Public health decision makers in developed and developing countries are exploring the idea of providing packages of health checks at specific times during a person's lifetime to detect and/or prevent life-threatening diseases such as diabetes, heart problems, and some cancers. Bundling together tests for different diseases has advantages for both health-care systems and patients. It can save time and money for both parties and, by associating health checks with life events such as childbirth, it can take advantage of a valuable opportunity to check on the overall health of individuals who may otherwise rarely visit a doctor. But money and other resources (for example, nurses to measure blood pressure) are always limited, even in wealthy countries, so decision makers have to assess the likely costs and benefits of packages of interventions before putting them into action.

**Why Was This Study Done?:**

Recent evidence suggests that women in developing countries would benefit from a once-in-a-lifetime screen for cervical cancer, a leading cause of cancer death for this population. If such a screening strategy for cervical cancer were introduced, it might provide a good opportunity to offer women other health checks, but it is unclear which interventions should be packaged together. In this study, the researchers have developed an analytic framework to identify an optimal package of health services to offer to women attending a clinic for their lifetime cervical cancer screen. Their model takes into account monetary limitations and possible shortages in trained personnel to do the health checks, and balances these constraints against the likely health benefits for the women.

**What Did the Researchers Do and Find?:**

The researchers developed a “mathematical programming” model to identify an optimal package of health services to be provided during a single visit. They then used their model to estimate the average costs and health outcomes per woman of various combinations of health interventions for 35- to 40-year-old women living in four regions of the world with high adult death rates. The researchers chose breast cancer, cardiovascular disease, depression, anemia caused by iron deficiency, and sexually transmitted diseases as health conditions to be checked in addition to cervical cancer during the single visit. They considered two ways—one cheap in terms of money and people; the other more expensive but often more effective—of checking for or dealing with each potential health problem. When they set a realistic budgetary constraint (based on the annual health budget of the poorest countries and a single health check per woman in the two decades following her reproductive years), the optimal health package generated by the model for all four regions included cervical cancer screening done by testing for human papillomavirus (an effective but complex test), treatment for depression, and screening or treatment for anemia. When a 50% shortage in general (for example, nurses) and specialized (for example, doctors) personnel time was also included, the health benefits of the package were maximized by using a simpler test for cervical cancer and by treating anemia but not depression; this freed up resources in some regions to screen for breast cancer or cardiovascular disease.

**What Do These Findings Mean?:**

The model described by the researchers provides a way to explore the potential advantages of delivering a package of health interventions to individuals in a single visit. Like all mathematical models, its conclusions rely heavily on the data used in its construction. Indeed, the researchers stress that, because they did not have full data on the effectiveness of each intervention and made many other assumptions, their results on their own cannot be used to make policy decisions. Nevertheless, their results clearly show that the packaging of multiple health services during a single visit has great potential to maximize health gains, provided the right interventions are chosen. Most importantly, their analysis shows that in the real world the shortage of personnel, which has been ignored in previous analyses even though it is a major problem in many developing countries, will affect which health conditions and specific interventions should be bundled together to provide the greatest impact on public health.

**Additional Information.:**

Please access these Web sites via the online version of this summary at http://dx.doi.org/10.1371/journal.pmed.0030434.g001.

- The World Health Organization has information on choosing cost-effective health interventions and on human resources for health
- The American Cancer Society offers patient information on cervical cancer
- The Alliance for Cervical Cancer Prevention includes information about cervical cancer prevention programs in developing countries

## Introduction

Although substantial public health efforts are directed toward improving the health and birth outcomes of pregnant women [1], nearly two-thirds of healthy years lost by women in developing countries are caused by problems other than reproductive health [2]. A disease that is particularly relevant to women in developing countries is cervical cancer, which remains a leading cause of cancer death [3]. Cervical cancer is preventable by identifying (by Pap smears) and treating precancerous lesions before they progress to cancer. Mortality from this cancer has been reduced in countries able to sustain cervical screening programs, but these kinds of programs have been difficult to implement in poor countries because of the laboratory and technical expertise required, the multiple visits required for screening, diagnosis, and treatment, and the need for repeated screening at regular intervals. There is increasing evidence that cervical cancer screening once or twice between ages 35 and 45 years, using screening tests that both require less technical expertise and can be embedded in strategies that enhance the linkage between screening and treatment, would be beneficial to the health of patients, cost-effective, and feasible [4–8].

For countries considering the introduction of single lifetime screening strategies, the point of contact between a woman and the health care system during the screening visit represents an opportunity to offer other health interventions, increasing access to essential services, and reducing patient time and travel costs. The design of such a package of services requires careful consideration of both the potential health gains achievable with different interventions and the resources available to support them. Although an obvious constraint is the monetary resources available to fund health programs, the shortage of human resources is increasingly acknowledged as an even more formidable obstacle [9–11].

While other analyses have evaluated the costs and benefits of packaging health services in developing country settings [12], none have incorporated resource constraints other than the lack of funds. Motivated by the momentum associated with the international interest in single lifetime cervical cancer screening strategies, we developed an analytic framework to identify an optimal package of health services that explicitly takes into consideration both monetary and human resource constraints. We demonstrate this framework by presenting a simplified example, which includes a select group of diseases, interventions, and constraints that are relevant among women in four resource-poor regions of the world.

## Methods

### Analytic Overview

We developed a binary integer programming (IP) model capable of identifying an optimal package of health services to be provided during a single visit for a particular target population. We selected an IP model to make the explicit assumption that a chosen intervention must cover all eligible individuals. Inputs to the IP model are derived using state-transition models, which simulate the effects of an intervention on the natural history of disease and compute lifetime costs and health benefits associated with each intervention. The IP algorithm considers all possible combinations of interventions and identifies the package of services that simultaneously satisfies the objective function (e.g., maximize health benefits) while adhering to the specified constraints. The binary aspect requires that interventions are either fully implemented (1) or not implemented at all (0).

### Data Requirements and Synthesis

Data on incidence, prevalence, and mortality for each health condition are synthesized using state-transition models. Individuals enter each disease-specific model at a particular age, transition through a series of health states reflecting the natural history of each disease, and face competing mortality rates from other causes. For each disease, multiple competing choice interventions with different levels of complexity, effectiveness, and costs may be considered. Depending on the perspective of the analysis, relevant costs are included in the model; for example, with a health systems perspective, direct medical costs associated with each intervention option would be included. Data on the effectiveness of screening and/or treatment are entered into the model. The models are then used to generate estimates of the average per-woman lifetime costs and health outcomes associated with each intervention option. Disease-specific outcomes are translated into standardized measures of lost years of healthy life, expressed as disability-adjusted life years (DALYs) [13]. DALYs include both years of life lost due to premature mortality and years lived in less than perfect health, adjusted for severity using a “disability weight” that can range from 0 (no disability) to 1 (complete disability). DALYs are estimated with and without the intervention, and the difference between the two measures represents the DALYs averted by the intervention.

In order to account for human resource requirements, we considered the personnel time required for the main initial visit at which women receive the package of services, as well as for follow-up diagnostic procedures for women who require them. Providers can be differentiated by level of training, education, and availability, such as “specialized” to indicate personnel with advanced skills or training (e.g., physicians and laboratory specialists), or “general” to indicate those with less specialized training (e.g., nurse midwives and auxiliary medical staff).

### IP Model

**Figure 1:**
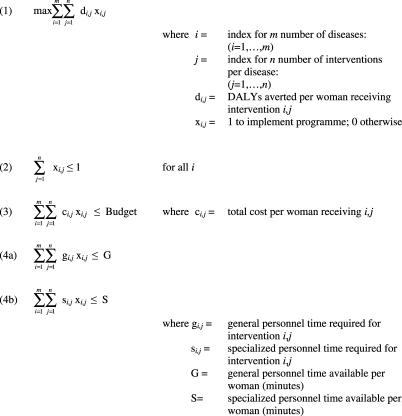
Equations of the Integer Programming Model The IP model consists of the objective function, which maximizes DALYs averted through the health interventions (equation 1); the constraint of choosing no more than one intervention option per disease (equation 2); a budget constraint, expressed as a per-woman lifetime health expenditure (equation 3); and human resource constraints, limiting the available general (equation 4a) and specialized (equation 4b) personnel time separately.

As shown in Figure 1, the IP model consists of the objective function, which maximizes DALYs averted through the health interventions (equation 1), the constraint of choosing no more than one intervention option per disease (equation 2), a budget constraint, expressed as a per-woman lifetime health expenditure (equation 3), and human resource constraints, limiting the available general (equation 4a) and specialized (equation 4b) personnel time separately.

### A Simplified Example

In order to demonstrate how one might synthesize data and conduct such an analysis, we elected to present a simplified example using our motivation for leveraging single lifetime cervical cancer screening to package other health services. We recognize and emphasize to the reader that the full set of data required for a comprehensive policy analysis were not available, but that we use this example to serve as a pedagogical tool and an application of the methods. It is important to note that provision of an intervention in a single visit refers to the delivery of the initial component of the intervention (e.g., screening test) but does not require that the follow-up of the identified subset of patients who require subsequent treatment occur in that same visit (see details below, and in Text S1).

Our target population was women between the ages of 35 and 40 years in four regions of the world with the highest burden of diseases and the highest child and adult mortality rates [2]. As categorized by the World Health Organization (WHO), these included Africa (AFR-D,E), Americas (AMR-D), Eastern Mediterranean (EMR-D), and Southeast Asia (SEAR-D). The designations D and E refer to WHO mortality strata that define states within the accompanying countries or regions; D indicates states with high child mortality and high adult mortality, and E indicates states with high child mortality and very high adult mortality. Future studies may contextualize the analysis to the regional, country, or clinic level, provided data are available.

In addition to cervical cancer, we chose five health conditions that affect women after their childbearing years, and for which data on burden of disease were available from the published literature (Table 1) [2]. These additional health conditions included breast cancer, cardiovascular disease (CVD), depression, iron deficiency anemia, and sexually transmitted diseases (STDs, excluding HIV). In this example, we excluded diseases already being targeted by major initiatives, such as AIDS, tuberculosis, and malaria [14].

**Table 1:**
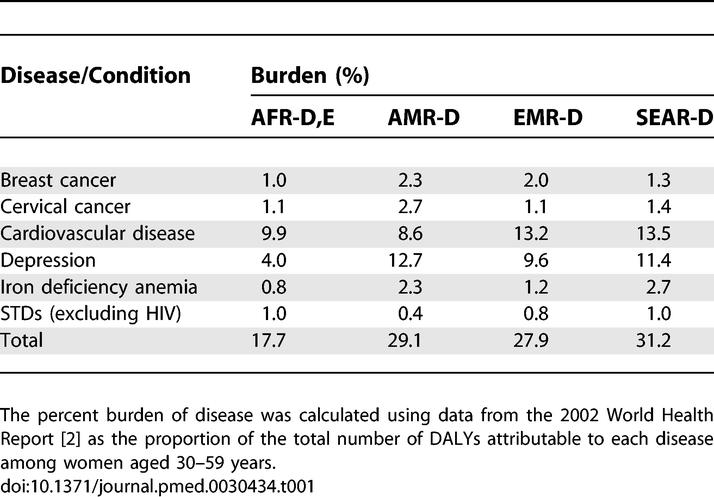
Percent Burden of Disease among Women Aged 30–59 Years Attributable to Selected Conditions

Based on the best available data, we included two intervention options for each disease (Table 2). In general, the more costly of the two was more effective and more technically complex to provide examples of interventions with differing levels of intensity. These particular interventions were selected because of the evidence of potential efficacy in the population of interest [15–28]. It is worth noting that we knowingly included interventions that have conventionally not been provided at a single point in time (e.g., breast cancer screening). This choice was made in part because published efficacy data for alternative interventions targeted to women in this age group were limited, and in part because the nature of our example is intended to serve as a demonstration of the methods. Models were constructed using TreeAge Pro software (Williamstown, Massachusetts, United States), and all costs were expressed in 2001 international dollars ($) to allow for comparisons across regions. We approximated the personnel time required for the main initial visit and for follow-up diagnostic procedures on the basis of evidence from the literature and expert opinion. Further details of model inputs and assumptions, intervention costs and effects, and personnel time requirements are included in Text S1. Table 3 shows the per-woman lifetime costs and DALYs averted estimated by the models by intervention and region.

**Table 2:**
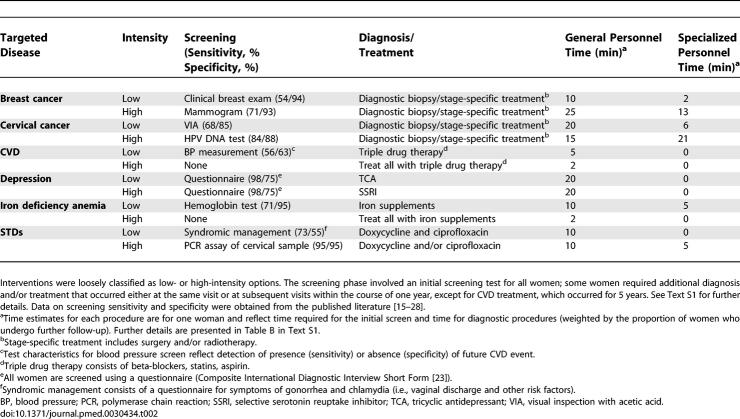
Selected Interventions

**Table 3:**
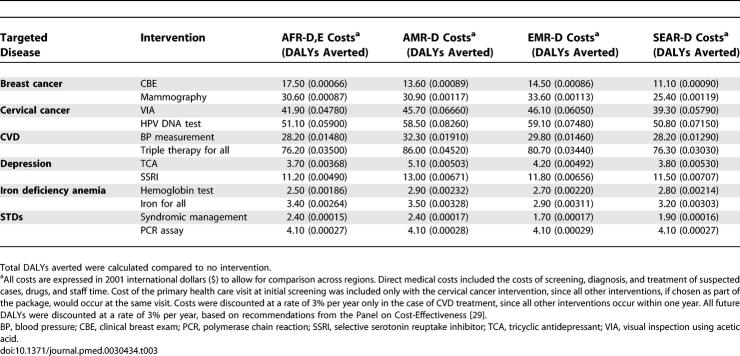
Total Per-Woman Lifetime Costs and DALYs Averted

In keeping with the motivation outlined in our introduction, we included an additional constraint of a mandatory cervical cancer screen at age 40 (see Text S1 for corresponding IP model equations). We set our initial budget to $75 per woman, based on the annual health care expenditure of $5 per capita in the poorest countries [30], presuming that this single lifetime visit was targeted to women only once within two decades following their reproductive years and discounting future costs at 3% per year. Because we lacked region-specific data on available personnel time, for each type of personnel we calculated the time required for each disease, which was averaged between the low- and high-intensity intervention options. Constraints were expressed as fractions of the total amounts of general and specialized personnel times required to implement one intervention for each disease. In this particular example, we applied an arbitrary constraint of 50% on both general and specialized personnel times, and then on specialized personnel time only to explore the influence of relative availability among different personnel types. We also explored the implications of changing the budget.

## Results

Table 4 shows the selection of interventions from our example of a one-time cervical cancer screening visit under different sets of constraints. When we constrained only the budget, interventions for cervical cancer, depression, and iron deficiency anemia were included in all four regions, while interventions for breast cancer and CVD were not (results for budget-only constraint). In AFR-D,E and SEAR-D, PCR testing for sexually transmitted diseases was also selected for the package. With the exception of hemoglobin testing for iron deficiency in AMR-D, the more costly option for each disease-specific intervention was chosen.

**Table 4:**
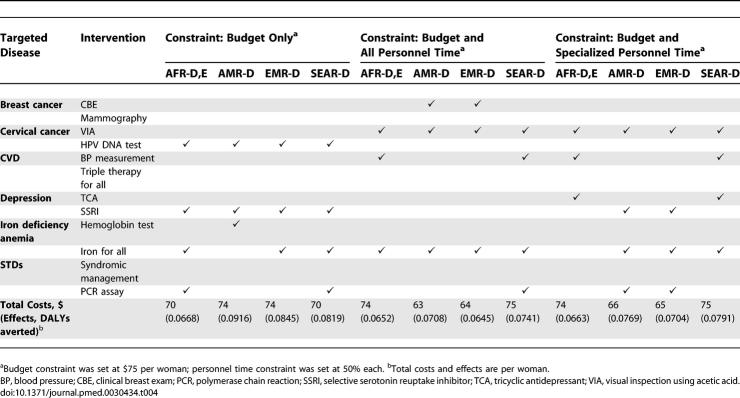
Results of IP Analysis: Package of Health Services under Constraints of Budget and Personnel Time

When we introduced a 50% shortage of both general and specialized personnel time (results on budget with all personnel constraints), the less intensive strategy for cervical cancer was chosen (visual inspection with acetic acid), and treatment for depression was excluded, allowing for the adoption of other interventions, such as blood pressure screening for CVD in AFR-D,E and SEAR-D, and clinical breast examination in AMR-D and EMR-D. When we restricted only specialized personnel, a particularly scarce resource in developing country settings (results on budget and 50% specialized personnel time), the less intensive strategy for cervical cancer was again chosen. Because we assumed that there would be no shortage of general personnel in this scenario, one of the intervention options for depression was selected in each of the four regions; although CVD interventions met the personnel time requirements, they exceeded the budget constraint. For the most part, iron supplementation for all women was chosen as part of the package under this scenario, as well as the other two scenarios.

**Figure 2:**
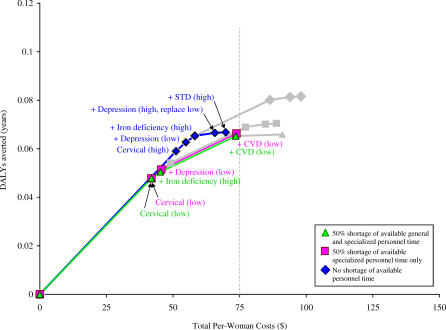
Packages of Interventions under a Lifetime Budget of $75 per Woman in AFR-D,E Each curve shows the order in which interventions are added (or substituted) when the budget is set at $75 per woman under the different scenarios of personnel time constraints: no personnel time constraints (blue), 50% constraint on specialized personnel time only (pink), and 50% constraint on both general and specialized personnel time (green). Curves in gray represent data from alternative lifetime budget set at $100 per woman (Figure 3). The dotted line represents the budget constraint.

**Figure 3:**
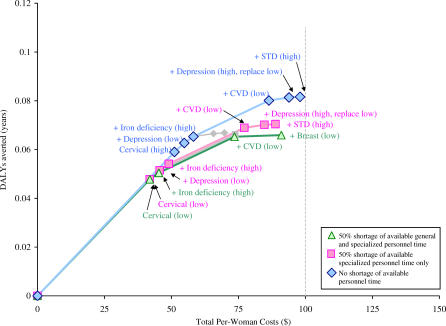
Packages of Interventions under a Lifetime Budget of $100 per Woman in AFR-D,E Each curve shows the order in which interventions are added (or substituted) when the budget is set at $100 per woman under the different scenarios of personnel time constraints: no personnel time constraints (blue), 50% constraint on specialized personnel time only (pink), and 50% constraint on both general and specialized personnel time (green). Curves in gray represent data from alternative lifetime budget set at $75 per woman (Figure 2). The dotted line represents the budget constraint.

Figure 2 specifies the order in which interventions are added (or substituted) for the particular budget of $75 per woman in AFR-D,E. The introduction of personnel time constraints results in the adoption of the less intensive options for cervical cancer and CVD. The similarity of the optimal package under both scenarios of personnel constraints suggests that the relative mix of general versus specialized personnel has little influence on the package itself. When the budget was increased to $100 per woman (Figure 3), blood pressure screening for CVD was included in all scenarios. The packages with personnel constraints differed from the package that would be delivered with only the budget constraint, and they produced lower overall benefits, primarily due to the adoption of the less intensive option for cervical cancer. Furthermore, the type of personnel constraint had a greater influence on the contents of the package. When the budget was decreased to $50 per woman (results not shown), the personnel time constraints no longer influenced the contents of the package.

## Discussion

In our simplified analysis, we considered packaging interventions that may be delivered in a single contact in order to maximize the potential burden of disease averted for a relatively neglected segment of the population, subject to two important resource constraints. We found that multiple interventions were selected to be included in a package of health services during a single cervical cancer screening visit. When only a budget constraint was considered, these interventions included treatment for depression, and screening or treatment for iron deficiency anemia and STDs (in two regions). With the additional constraints on general and specialized personnel, the contents of the optimal package shifted and included the low-intensity intervention option for cervical cancer, allowing for the inclusion of other interventions in some regions, such as screening for breast cancer and cardiovascular disease.

The goal of this analysis was to demonstrate an analytic framework that can be used to explore potential advantages of bundling interventions to be delivered in a single visit. We encourage a shift from the traditional mindset of assessing costs and benefits of interventions for reducing mortality and morbidity attributable to a single disease to exploring what synergies might be achievable by thinking across disease burdens.

Many countries, both developed and developing, have explored the concept of packaging health services at select, opportune moments in an individual's life, such as the perinatal period for both woman and child. The World Bank has identified as many as 18 countries that have made attempts to design a package of essential health services, citing 12 countries that have engaged in formal quantitative analyses to inform these decisions, such as cost-effectiveness analysis [12]. Our analysis differs from a cost-effectiveness analysis in that we explicitly consider a human resource constraint in addition to a monetary constraint. Other linear programming models that have been used to optimize resource allocation in the context of health have generally considered only budget constraints [31–36].

Increasingly, decision makers are interested in constraints such as specialized personnel [9–11] as well as affordability. Our framework presents one method of formally and explicitly taking into account nonmonetary constraints when considering a package of services. The results from our example suggest that the addition of personnel constraints was influential under both budget limits. For example, although human papillomavirus (HPV) DNA testing was the preferred option for cervical cancer screening when only the budget was considered, an alternative screening option with fewer requirements for technical personnel was chosen when we placed constraints specifically on the specialized personnel time. The selection of the simpler strategy actually then allowed for the inclusion of interventions for other diseases, such as screening and treatment for CVD and breast cancer. Although a stylized example, this particular result provides insight into the potential value of investments in rapid and simple diagnostics for developing countries, such as the rapid HPV test [37].

There are several limitations to both our analytic framework and stylized example. First, we did not incorporate every potential advantage of packaging services. Since we did not include transportation costs, the potential travel-related savings from avoiding multiple visits were not included. We also did not include all shared resources among interventions. There may be as-yet unidentified negative consequences of packaging, and these were also not included [38]. We did not consider other constraints that may be influential (e.g., facilities, roads, infrastructure); however, the analytic framework flexibly allows for future analyses to include such resource requirements.

In our simplified example, we focused on a narrow group of health conditions and interventions and examined a limited range of budget and personnel constraints. Although infinite sets of diseases and interventions, and of budget and personnel constraints, are possible, we arbitrarily chose the current set to identify and illustrate key themes. For a particular country wishing to employ these methods to inform local decision making, in addition to identifying the relevant nonmonetary constraints for their setting, they may choose a predefined set of criteria to select the candidate diseases and interventions. Further, the DALY is only one metric of health outcomes, and limitations in the methods and data underlying the calculations of DALYs have been discussed elsewhere [39,40]; in future analyses, alternative measures of health outcomes, such as life expectancy, may be reflected.

Our choice to present the specific example as a pedagogical tool to demonstrate the use of these methods—rather than as a policy analysis—reflects the importance we place on explicitly acknowledging the data limitations. For this example there were substantial data gaps for effectiveness, costs, and personnel time estimates associated with all interventions, and therefore simplifying assumptions were necessary. Even when estimates of efficacy were available, the studies from which they were extrapolated were conducted mainly in developed country settings, and for some variables (e.g., treatment efficacy) a good deal of uncertainty and variability among studies existed. For our example, we synthesized the best available data from multiple sources in the literature in order to demonstrate how the framework can be applied to a particular problem. A notable advantage of the model is that for future analyses, model parameters can easily be contextualized with country-specific data.

These methods provide a framework to enhance a decision maker's ability to simultaneously consider costs, benefits, and important nonmonetary constraints. We encourage a shift from the traditional mindset of assesing costs and benefits of interventions that reduce mortality and morbidity attributable to a single disease to exploring what synergies might be achievable by thinking across disease burdens. As illustrated by our simplified example, the packaging of multiple health services during a one-time visit has the potential to increase health gains, and constraints such as specialized personnel shortages have the potential to influence the contents of the package. With the mobilization of health services for conditions such as cervical cancer, AIDS, tuberculosis, and malaria [14], this framework has real-world relevance and can be applied to multiple situations to leverage the contact between the health care system and those with limited opportunities to receive health care.

## Supporting Information

Text S1 Technical Appendix (558 KB DOC)

## Abbreviations

AFR: Africa
AMR: Americas
CVD: cardiovascular disease
DALY: disability-adjusted life year
EMR: Eastern Mediterranean
HPV: human papillomavirus
IP: integer programming
SEAR: Southeast Asia
STD: sexually transmitted disease

## References

[pmed-0030434-b001] Goodburn E, Campbell O (2001). Reducing maternal mortality in the developing world: Sector-wide approaches may be the key. BMJ.

[pmed-0030434-b002] World Health Organization (2002). The World Health Report 2002: Reducing risks, promoting healthy life.

[pmed-0030434-b003] Yang BH, Bray FI, Parkin DM, Sellors JW, Zhang ZF (2004). Cervical cancer as a priority for prevention in different world regions: An evaluation using years of life lost. Int J Cancer.

[pmed-0030434-b004] Denny L, Kuhn L, De Souza M, Pollack AE, Dupree W (2005). Screen-and-treat approaches for cervical cancer prevention in low-resource settings: A randomized controlled trial. JAMA.

[pmed-0030434-b005] Blumenthal PD, Gaffikin L (2005). Cervical cancer prevention: Making programs more appropriate and pragmatic. JAMA.

[pmed-0030434-b006] Goldie SJ, Gaffikin L, Goldhaber-Fiebert JD, Gordillo-Tobar A, Levin C (2005). Cost-effectiveness of cervical cancer screening in five developing countries. N Engl J Med.

[pmed-0030434-b007] Schiffman M, Castle PE (2005). The promise of global cervical-cancer prevention. N Engl J Med.

[pmed-0030434-b008] Goldie SJ, Kuhn L, Denny L, Pollack A, Wright TC (2001). Policy analysis of cervical cancer screening strategies in low-resource settings: Clinical benefits and cost-effectiveness. JAMA.

[pmed-0030434-b009] Nullis-Kapp C (2005). Health worker shortage could derail development goals. Bull World Health Organ.

[pmed-0030434-b010] Hongoro C, McPake B (2004). How to bridge the gap in human resources for health. Lancet.

[pmed-0030434-b011] Kober K, Van Damme W (2004). Scaling up access to antiretroviral treatment in southern Africa: Who will do the job?. Lancet.

[pmed-0030434-b012] Bobadilla JL, Cowley P (1995). Designing and implementing packages of essential health services. J Int Development.

[pmed-0030434-b013] Murray CJ (1994). Quantifying the burden of disease: The technical basis for disability-adjusted life years. Bull World Health Organ.

[pmed-0030434-b014] Brugha R, Donoghue M, Starling M, Ndubani P, Ssengooba F (2004). The Global Fund: Managing great expectations. Lancet.

[pmed-0030434-b015] Barton MB, Harris R, Fletcher SW (1999). The rational clinical examination. Does this patient have breast cancer? The screening clinical breast examination: Should it be done? How?. JAMA.

[pmed-0030434-b016] Bobo JK, Lee NC, Thames SF (2000). Findings from 752,081 clinical breast examinations reported to a national screening program from 1995 through 1998. J Natl Cancer Inst.

[pmed-0030434-b017] Humphrey LL, Helfand M, Chan BK, Woolf SH (2002). Breast cancer screening: A summary of the evidence for the U.S. Preventive Services Task Force. Ann Intern Med.

[pmed-0030434-b018] Denny L, Kuhn L, Pollack A, Wainwright H, Wright TC (2000). Evaluation of alternative methods of cervical cancer screening for resource-poor settings. Cancer.

[pmed-0030434-b019] Kuhn L, Denny L, Pollack A, Lorincz A, Richart RM (2000). Human papillomavirus DNA testing for cervical cancer screening in low-resource settings. J Natl Cancer Inst.

[pmed-0030434-b020] Sankaranarayanan R, Basu P, Wesley RS, Mahe C, Keita N (2004). Accuracy of visual screening for cervical neoplasia: Results from an IARC multicentre study in India and Africa. Int J Cancer.

[pmed-0030434-b021] Sankaranarayanan R, Budukh AM, Rajkumar R (2001). Effective screening programmes for cervical cancer in low- and middle-income developing countries. Bull World Health Organ.

[pmed-0030434-b022] Sankaranarayanan R, Chatterji R, Shastri SS, Wesley RS, Basu P (2004). Accuracy of human papillomavirus testing in primary screening of cervical neoplasia: Results from a multicenter study in India. Int J Cancer.

[pmed-0030434-b023] Patten SB, Brandon-Christie J, Devji J, Sedmak B (2000). Performance of the Composite International Diagnostic Interview Short Form for major depression in community and clinical samples. Chronic Dis Can.

[pmed-0030434-b024] Sari M, de Pee S, Martini E, Herman S, Sugiatmi (2001). Estimating the prevalence of anaemia: A comparison of three methods. Bull World Health Organ.

[pmed-0030434-b025] Law MR, Wald NJ, Morris JK (2004). The performance of blood pressure and other cardiovascular risk factors as screening tests for ischaemic heart disease and stroke. J Med Screen.

[pmed-0030434-b026] Pettifor A, Walsh J, Wilkins V, Raghunathan P (2000). How effective is syndromic management of STDs?: A review of current studies. Sex Transm Dis.

[pmed-0030434-b027] Watson EJ, Templeton A, Russell I, Paavonen J, Mardh PA (2002). The accuracy and efficacy of screening tests for Chlamydia trachomatis: a systematic review. J Med Microbiol.

[pmed-0030434-b028] Schwebke JR, Sadler R, Sutton JM, Hook EW (1997). Positive screening test for gonorrhea and chlamydial infection fail to lead consistently to treatment of patients attending sexually transmitted disease clinic. Sex Transm Dis.

[pmed-0030434-b029] Gold MR, Siegel JE, Russel LB, Weinstein MC (1996). Cost-effectiveness in health care and medicine.

[pmed-0030434-b030] Musgrove P, Zeramdini R, Carrin G (2002). Basic patterns of national health expenditures. Bull World Health Organ.

[pmed-0030434-b031] Stinnett AA, Paltiel AD (1996). Mathematical programming for the efficient allocation of health care resources. J Health Econ.

[pmed-0030434-b032] Earnshaw SR, Dennett SL (2003). Integer/linear mathematical programming models: A tool for allocating healthcare resources. Pharmacoeconomics.

[pmed-0030434-b033] Chen MM, Bush JW (1976). Maximizing health system output with political and administrative constraints using mathematical programming. Inquiry.

[pmed-0030434-b034] Wang LY, Haddix AC, Teutsch SM, Caldwell B (1999). The role of resource allocation models in selecting clinical preventive services. Am J Manag Care.

[pmed-0030434-b035] Tao G, Abban BK, Gift TL, Chen G, Irwin KL (2004). Applying a mixed-integer program to model re-screening women who test positive for C. trachomatis infection. Health Care Manag Sci.

[pmed-0030434-b036] Granata AV, Hillman AL (1998). Competing practice guidelines: Using cost-effectiveness analysis to make optimal decisions. Ann Intern Med.

[pmed-0030434-b037] Lorincz A, Eder P, Sellors J, Nazarenko (2004). Creation of an HPV test for developing countries. 2004 21–23 October; EUROGIN 2004 International Expert Meeting, Nice.

[pmed-0030434-b038] Bryce J, Black RE, Walker N, Bhutta ZA, Lawn JE (2005). Can the world afford to save the lives of 6 million children each year?. Lancet.

[pmed-0030434-b039] Williams A (1999). Calculating the global burden of disease: Time for a strategic reappraisal?. Health Econ.

[pmed-0030434-b040] Murray CJ, Lopez AD (2000). Progress and directions in refining the global burden of disease approach: A response to Williams. Health Econ.

