# Supplementary material for: Packaging Health Services When Resources Are Limited: The Example of a Cervical Cancer Screening Visit
Source: PLoS Med. 2006 Nov 14;3(11):e434. doi: 10.1371/journal.pmed.0030434 (PMC1635742; doi:10.1371/journal.pmed.0030434)
Supplement: Supplementary file 1 [file pmed.0030434.sd001.doc]

Packaging Health Services with Consideration of Limited Monetary and Human Resources:

The Example of a Single Cervical Cancer Screening Visit

Jane J. Kim, Joshua A. Salomon, Milton C. Weinstein, Sue J. Goldie

**Technical Appendix for Simplified Example**

*IP Model*

As shown in Figure A, the IP model consists of (1) the objective function, which maximizes DALYs averted through the health interventions, (2) the constraint of choosing no more than one intervention option per disease, and (3) the mandatory inclusion of cervical cancer screening at age 40.

In the first analysis, we included only a budget constraint (4), expressed as a per-woman lifetime health expenditure. We set our initial budget to $75 per woman, based on the annual health care expenditure of $5 per capita in the poorest countries [1], presuming that this single lifetime visit was targeted to women only once within two decades following their reproductive years and discounting future costs at 3% per year.

We then added human resource constraints to the analysis (5a, 5b), limiting the available general and specialized personnel time separately. For each type of personnel, we calculated the time required for each disease, which was averaged between the low and high intensity intervention options. Constraints were expressed as fractions of the total amounts of general and specialized personnel times required to implement one intervention for each disease.

*State-Transition Models, Interventions, and Personnel Time for Each Health Condition*

###### Breast cancer (Figure B)

The breast cancer model contains one healthy state and local, regional, and distant cancer states, consistent with the U.S. Surveillance Epidemiology and End Results (SEER) classification system for cancer stages [2]. Women face an annual risk of developing breast cancer at the local stage, which can progress to regional and distant stages over time; detection of cancer can occur through either symptoms or screening. Age- and region-specific estimates of breast cancer incidence were obtained from the International Agency for Research on Cancer (IARC) [3]. For lack of data in each of these regions, we assumed that 5-year survival rates are similar to those of women in the U.S.

In the absence of screening, we adjusted symptom detection rates and progression of cancer stages in order to match the stage distribution of cancer reported by a clinic in India, where unscreened women showed up with symptoms of cancer [4]. In addition, once we superimposed the interventions onto the disease model, we further attempted to calibrate the model to observed data reported from screening or treatment studies that evaluated more frequent intervals than once per lifetime. For instance, we conducted an analysis of annual breast cancer screening using mammography and adjusted parameters of the model (i.e., treatment effectiveness) such that mortality reductions projected from our model matched outcomes reported in clinical trials [5-7], and stage distribution of cancer matched current estimates observed in the U.S. [2]. We also assessed model corroboration by comparing life expectancy gains associated with annual screening with that of other published modeling studies [8,9]. We then used this adjusted model to estimate the costs and DALYs associated with a single screen (with either clinical breast exam or mammogram) at the age of 40.

Estimates of sensitivity and specificity for the breast cancer screening strategies were obtained from meta-analyses and literature reviews of clinical trials primarily conducted in the U.S. [5-7]. Although there is substantial debate over the test performance of mammography among younger women and clinical breast examination, in general, we used estimates within the reported ranges found in the literature. Further, all of the studies examined the efficacy of either screening modality using frequent screening intervals, not a one-time screen, making accurate estimates of test sensitivity and specificity challenging. For simplicity, we assumed that biopsy is perfect for diagnosing cancer; however, studies estimate the rate of benign breast disease to be quite high among women with positive mammography. Disability weights were estimated from health state utilities from the literature, and are assumed to be 0.20 for local cancer, 0.37 for regional cancer, and 0.71 for distant cancer [10,11]. Because we do not have region-specific costs, we capitalized on the availability of primary data on cervical cancer screening costs from several regions of the world, including countries from the four regions of the current study. We estimated the relative cost of a cervical screen in each region compared to the U.S., and then used these proportions to scale down U.S. costs for breast cancer screening and treatment obtained from published studies [8-10,12,13].

In terms of personnel time, for the clinical breast exam (CBE), we assumed that a nurse (or other general personnel) needs 10 minutes to complete examination of both breasts, based on a published literature review of the effectiveness of CBE screening [5]. For the mammography screen, each woman spends 5 minutes with an administrative staff in preparation for the mammogram, and the mammogram itself is assumed to take 20 minutes with a general technician. We assumed that a radiologist then takes 10 minutes with each mammogram to interpret the results. From the disease model, we obtained an estimate of the proportion of women who gets a positive screen (6% with CBE and 7% with mammogram). These women are assumed to receive a biopsy, which requires, on average, 20 minutes from a surgeon to obtain the sample, 10 minutes from a pathologist to interpret the sample, and 5 minutes from quality control personnel per biopsy sample. Total time for CBE and mammography screen reflect these corresponding proportions of women who receive biopsy.

###### Cervical cancer (Figure C)

For the cervical cancer model, we used a previously developed model [14-17] that includes monthly risk of acquisition and clearance of HPV infection, and progression and regression from low-grade and high-grade precancerous lesions, classified as cervical intraepithelial neoplasia, grades 1 (CIN 1) and 2,3 (CIN 2,3), respectively, and development of invasive cancer. Estimates of lifetime risk of cervical cancer in each of the four regions were obtained from the IARC and were used as targets for calibration [3], assuming that screening is infrequent or non-existent. We adjusted two parameters until the lifetime cancer risk from the model was similar to the IARC estimates in each region: the age-specific incidence of HPV infection, which reflects onset of sexual activity and can vary by region, and progression from high-grade lesions (CIN 2,3) to cancer, an unobservable parameter. Across all four regions, once in a lifetime screening with either VIA or HPV DNA testing reduced lifetime risk of cervical cancer by 25-31%, consistent with previously reported analyses [15,17,18].

Estimates of VIA and HPV DNA test performance were obtained from published reports of screening programs in resource-poor settings, such as India and Africa [19-23]. VIA test characteristics in particular are controversial and have been shown to vary across different settings, but we used values that fall within a relevant range of pooled estimates from multi-site cross-sectional studies. Test sensitivity for both tests was defined as the probability of testing positive among women with CIN or cancer. We assumed that 20% of all women screened using either VIA or HPV test are referred to a secondary-level facility because of sample inadequacy, difficulty in making a diagnosis, or difficulty in immediate treatment of detected precancerous lesions. Treatment for cancers and removal of any precancerous lesions are assumed to be 100% effective in this analysis. Disability weights were based on stage-specific cancer utilities from the published literature, and are 0.32, 0.44, 0.52 for local, regional, and distant cancer, respectively [24]. Cervical cancer screening and treatment costs were based on primary data from several regions of the world, including countries from the four regions of the current study [15,17,25].

For cervical cancer screening, we assumed that each woman spends 5 minutes of preparatory time with administrative personnel for either of the screening methods. Visual inspection of the cervix with application of acetic acid is assumed to take, on average, 15 minutes and can be performed by a nurse practitioner. For HPV DNA testing, procedure time to obtain a cervical sample is assumed to be 10 minutes (also performed by a nurse). Sample processing and interpretation by a highly-trained laboratory technician takes an average of 10 minutes, and quality control takes an additional 5 minutes, consistent with published microcosting estimates of time required to process, interpret and quality control for cytology samples [26]. Colposcopy and biopsy procedure time for those women who require further diagnostic workup is 20 minutes by a physician, and pathology and quality control require 5 minutes of time each per biopsy sample.

###### CVD (Figure D)

Cardiovascular disease events include stroke and acute myocardial infarction (MI). We assumed that women who experience either stroke or MI are subject to an immediate risk of death, after which they are susceptible to repeat events at higher rates. We obtained age- and region-specific incidence data for stroke from literature that was published as part of the Global Burden of Disease 2000 report [27]. Rates of first MI, recurrent MI and stroke, and death for untreated MI were obtained from a review of published cohort studies [28], and rates of death for first-ever stroke were from a prospective study in Australia [29].

Without intervention, our model predicts lifetime risk of stroke and MI that are very close to those published in the literature (12% versus 13% for stroke, and 15% versus 13% for MI) [28]. With triple drug therapy (i.e., beta-blockers, statins, and aspirin), we adjusted treatment effectiveness in order to match clinical trial outcomes of reduction in stroke and MI events. Triple drug therapy was adjusted to reduce CVD events by 75% per year for the duration of treatment. This estimate of effectiveness is consistent with reports of efficacy from a literature review that summarizes the risk reduction of stroke and MI events associated with different types and combinations of drug therapies from multiple clinical trials [28].

We used systolic blood pressure (SBP) >140 mmHG as the positivity criterion for blood pressure screening. Test sensitivity of blood pressure measurement is defined as the probability of having SBP >140 mmHG among those who experience a CVD event in that year; test specificity is the probability of having SBP <140 mmHG among those who do not experience a CVD event in that year. These measures of blood pressure test performance were based on blood pressure measurements of individuals who had either a stroke or MI over a 10 year period, and were obtained from a recent Health Technology Assessment report [28]. The blood pressure reading itself is assumed to measure blood pressure perfectly. In the screening strategy, risk reduction associated with triple drug therapy is only applied to women who have a reading of SBP >140 mmHG, including both true positive and false positive cases. In the treat all strategy, the reduction of risk for a CVD event is applied to the general population. Risk reduction is assumed for all 5 years of the treatment duration.

Costs of blood pressure screening and distribution of triple drug therapy to all women consist only of lower-level personnel time. Costs of treatment were obtained from the WHO-CHOICE (“Choosing Interventions that are Cost-Effective”) database [30] and include annual cost of beta-blockers, statins, and aspirin and one additional primary health care visit per year, for a duration of five years. Disability weights of 0.24 and 0.12 are assumed for stroke and MI states respectively, based on published estimates of health state utilities from stroke and MI survivors [31-33].

For the CVD intervention, blood pressure screening is assumed to take 5 minutes per woman, and the distribution of triple drug therapy to all women is assumed to take 2 minutes per woman, both by administrative personnel.

###### Depression (Figure E)

For the depression model, episodes of depression are assumed to be 6 months, after which recurrent episodes occur at higher rates. Data on annual incidence of depression were obtained from two published reports of depression in different parts of the world [34,35]. The rate of recurrent depressive episodes were based on a five-year prospective study of patients with major depression [36]. We assumed no excess mortality associated with depression. In the absence of treatment, our model predicts a lifetime risk of first episode of depression as 49%, close to the lifetime risk estimated at 45% for females up to the age of 70 [37].

For depression screening, performance of the Composite International Diagnostic Interview – Short Form (CIDI-SF) in detecting depression was evaluated in comparison to the full version of CIDI [38]. Treatment with TCA (i.e., 100 mg immipramine) is assumed to be 60% effective, and with SSRI (i.e., 20 mg fluoxetine) 80% effective in curing depressive episodes over the 6 month period of treatment, based on a meta-analysis of clinical trial data [39].

Screening cost of administering the CIDI-SF consists of 20 minutes of time with general personnel. The per-episode costs for depression treatment using TCA and SSRI were obtained from the WHO-CHOICE database [30]. We assumed that women with depression receive either TCA or SSRI, one primary health care visit, and one outpatient visit over the course of 6 months. The disability weight associated with a 6-month episode of depression is 0.60 [40].

The Composite International Diagnostic Interview Short Form (CIDI-SF) takes approximately 20 minutes to administer by a nurse or administrator, based on estimates of the full version of the CIDI [41]. No time with specialized personnel is required for either intervention.

###### Iron deficiency anemia (Figure F)

The iron deficiency anemia model includes a healthy and an anemia state. Women face an annual risk of developing anemia, and we assume that once a woman develops anemia, there is no spontaneous cure and no excess mortality. Annual incidence rates of iron deficiency anemia were based on a population-based national survey of iron deficiency anemia prevalence in China [42], and are assumed the same in all regions. In order to ensure that the incidence rates were estimated correctly, we projected prevalence of anemia for women at age 40 using the model and compared it against the prevalence reported in the survey (20-26% in the model versus 24% in the survey) [42]. With iron supplementation treatment for 6 months, the model output of DALYs averted were similar to the average DALY averted per woman reported in the WHO CHOICE database [30].

Screening for anemia is performed by assessment of capillary blood using a hemoglobin test; women with hemoglobin levels less than 119 g/L are considered to have anemia. Test performance in detecting cases of anemia was estimated from a study which used venous direct cyanmethaemoglobin as the gold standard [43]. Iron supplementation over 6 months is assumed to be 100% effective in curing prevalent cases of iron deficiency anemia, with no adverse reactions. Since most cases of anemia develop at pregnancy, we assumed that once a woman is treated for anemia at age 40, she is protected from developing anemia again in the future.

Cost of screening with the hemoglobin test in each region was obtained from a report on unit costs of health inputs in developing regions [44], and the cost of distributing iron supplements to all women include 2 minutes of lower-level personnel time. Costs of treatment with iron supplementation was obtained from the WHO-CHOICE database [30] and include cost of iron supplements for 6 months plus one additional primary health care visit. The disability weight associated with iron deficiency anemia reflects a weighted average of mild, moderate, and severe cases among adult women estimated from a survey in India (69%, 27%, and 4%, respectively) [42]. Disability weights are 0 for mild, 0.012 for moderate, and 0.090 for severe anemia [45], and therefore the weighted disability weight assumed in the model is 0.993.

The hemoglobin test requires 5 minutes for a nurse to draw capillary blood from a finger prick. Lab processing and interpretation by a general lab technician takes approximately 5 minutes, and quality assurance by a highly trained lab technician takes on average 5 minutes per blood sample. Distribution of iron supplements to all women is assumed to take 2 minutes per woman by administrative personnel.

###### Sexually transmitted diseases (STD) (Figure G)

For the STD model, two bacterial sexually transmitted infections, gonorrhea (GC) and chlamydia (CT), are modeled as independent events, and if left untreated, have a rate of progressing to acute pelvic inflammatory disease (PID), and possible sequelae (i.e., chronic pelvic pain, ectopic pregnancy, or infertility). Women with either STD or PID can have symptoms, and can experience a spontaneous cure. The probability of reinfection with either STD is higher in women with previous infections. We assumed that no excess mortality is associated with STD.

We obtained age- and region-specific incidence data for GC and CT from a published report of incidence and prevalence of curable STDs throughout the world [46,47]. Incidence of pelvic PID and sequelae were based on reviews of studies conducted at family planning clinics in both the U.S. and in developing countries [48,49]. Spontaneous clearance of STD was based on follow-up of women with only CT [50], but we assumed the same rate for women with GC. Reinfection rates of STD were based on a prospective study of adolescent girls previously infected with GC and/or CT [51].

Screening with syndromic management consists of a series of questions regarding symptoms of STDs, such as vaginal discharge and risk factors. Only symptomatic STD (and PID) are detectable using syndromic management, whereas cases of both symptomatic and asymptomatic STD are detectable using the PCR assay; women with symptomatic PID who are detected with syndromic management are treated ineffectively with the STD treatment regimen since PID requires more aggressive treatment. Performance of syndromic management in detecting cases of gonorrhea and/or chlamydia was estimated from a review of studies conducted primarily in low-resource settings, such as Indonesia, Cote d’Ivoire, and Zambia, among women who reported symptoms of vaginal discharge [52]. A proportion of women without vaginal discharge (29%) were also detected with syndromic management because they reported other risk factors, such as sexual history. Estimates of sensitivity and specificity of polymerase chain reaction (PCR) assay on cervical samples were based on a systematic review of studies conducted in Western Europe [53]. Treatment for GC is one dose of ciprofloxacin and for CT is two doses of doxycycline per day over a 7-day period. Although treatment with both drugs is considered to be 100% effective, compliance to treatment with ciprofloxacin is 100% and doxycycline is 80%, based on a study of adherence among patients who had tested positive for GC and/or CT at an STD clinic [54].

Cost of STD screening with syndromic management consists of 10 minutes of time with a lower-level staff; PCR lab test costs are based on a literature review of STD screening costs in Africa [55]. Costs for treatment were obtained from the WHO-CHOICE database [30] and include cost of drugs and one additional primary health care visit. Disability weights associated with GC and CT are 0.10 [46,56]. In addition, we reflect disability associated with untreated PID (disability weight of 0.42 for two weeks) and sequelae from PID (disability weight of 0.12) [46,56].

Screening with syndromic management is conducted by a nurse and takes on average 10 minutes of time. For the PCR assay test, a cervical sample can be procured during the cervical cancer screen (using either VIA or HPV DNA test). Specimen processing and interpretation, which can include testing for drug resistance, takes a general lab technician approximately 10 minutes. On average, each sample requires 5 minutes of time with a laboratory technician for quality assurance.

**Appendix References**

## Appendix Table A. Selected Model Parameters*

| **Variable** | **AFR-D,E** | **AMR-D** | **EMR-D** | **SEAR-D** |
| --- | --- | --- | --- | --- |
| Breast cancer [3] |  |  |  |  |
| Incidence rate | 0.00011-0.00067 | 0.00012-0.00084 | 0.00012-0.00062 | 0.00013-0.00086 |
| Local to regional cancer | 0.60 | 0.60 | 0.60 | 0.60 |
| Regional to distant cancer | 0.70 | 0.70 | 0.70 | 0.70 |
| Symptom detection (%) |  |  |  |  |
| Local | 15 | 15 | 15 | 15 |
| Regional | 50 | 50 | 50 | 50 |
| Distant | 95 | 95 | 95 | 95 |
| Cervical cancer [57-76] |  |  |  |  |
| Well to HPV | 0.0042-0.1268 | 0.0063-0.1901 | 0.0032-0.0951 | 0.0042-0.1268 |
| HPV clearance | 0.0336-0.3717 | 0.0336-0.3717 | 0.0336-0.3717 | 0.0336-0.3717 |
| HPV to CIN1 | 0.0553 | 0.0553 | 0.0553 | 0.0553 |
| CIN 1 regression | 0.0602 | 0.0602 | 0.0602 | 0.0602 |
| CIN 1 to CIN 2,3 | 0.0481 | 0.0481 | 0.0481 | 0.0481 |
| CIN 2,3 regression | 0.0060 | 0.0060 | 0.0060 | 0.0060 |
| CIN 2,3 to local cancer | 0.0361 | 0.0481 | 0.0271 | 0.0361 |
| Local to regional cancer | 0.30 | 0.30 | 0.30 | 0.30 |
| Regional to distant cancer | 0.49 | 0.49 | 0.49 | 0.49 |
| Symptom detection (%) |  |  |  |  |
| Local | 19 | 19 | 19 | 19 |
| Regional | 60 | 60 | 60 | 60 |
| Distant | 90 | 90 | 90 | 90 |
| CVD [27,28,77] |  |  |  |  |
| Incidence of stroke | 0.00040-0.05326 | 0.00040-0.02297 | 0.00020-0.02997 | 0.00013-0.03224 |
| Incidence of MI | 0.00001-0.01330 | 0.00001-0.01330 | 0.00001-0.01330 | 0.00001-0.01330 |
| Recurrence of stroke or MI | 0.10 | 0.10 | 0.10 | 0.10 |
| Depression [34-36] † |  |  |  |  |
| Incidence of first episode | 0.0080-0.0340 | 0.0101-0.0430 | 0.0110-0.0460 | 0.0110-0.0447 |
| Incidence of recurrent episode | 0.25 | 0.25 | 0.25 | 0.25 |

## Appendix Table A. Selected Model Parameters* (cont)

| **Variable** | **AFR-D,E** | **AMR-D** | **EMR-D** | | **SEAR-D** |
| --- | --- | --- | --- | --- | --- |
| Iron deficiency anemia [42] |  |  |  | |  |
| Incidence | 0.00250-0.00500 | 0.00250-0.00500 | 0.00250-0.00500 | | 0.00250-0.00500 |
| STD [46-51,56,78-80] ‡ |  |  |  | |  |
| Incidence of GC/CT | 0.00045-0.02700 | 0.00065-0.01870 | 0.00105-0.02120 | | 0.00105-0.02075 |
| Spontaneous cure | 0.40 | 0.40 | 0.40 | | 0.40 |
| Symptomatic STD | 0.25 | 0.25 | 0.25 | | 0.25 |
| Incidence of PID | 0.30 | 0.30 | 0.30 | | 0.30 |
| Symptomatic PID | 0.40 | 0.40 | 0.40 | | 0.40 |
| Incidence of sequelae § | 0.18 | 0.18 | 0.18 | | 0.18 |
| Relative risk of recurrent STD | 2.0 | 2.0 | 2.0 | | 2.0 |
| **Mortality rates** [2,29,77,81,82] **\|\|** |  |  |  | |  |
| All-cause | 0.0134-0.0164 | 0.0019-0.0056 | 0.0020-0.0067 | | 0.0038-0.0099 |
| Breast cancer | 0.0162-0.3604 | 0.0162-0.3604 | 0.0162-0.3604 | | 0.0162-0.3604 |
| Cervical cancer | 0.0041-0.0421 | 0.0041-0.0421 | 0.0041-0.0421 | | 0.0041-0.0421 |
| CVD |  |  |  | |  |
| Immediate, post-stroke or MI | 0.36 | 0.36 | 0.36 | | 0.36 |
| Subsequent, post-stroke or MI | 0.10 | 0.10 | 0.10 | | 0.10 |
| **Intervention costs** (2001 $) ¶ | | | | | |
| Wages (hourly) [44] |  |  | |  |  |
| Lower-level personnel | 3.92 | 3.92 | | 2.47 | 2.88 |
| Higher-level personnel | 14.24 | 12.52 | | 4.94 | 4.25 |
| Breast cancer [8-10,12,13] |  |  | |  |  |
| Clinical breast exam ** | 0.65 | 0.65 | | 0.41 | 0.48 |
| Mammogram screen | 15 | 16 | | 17 | 13 |
| Biopsy and excision | 191 | 204 | | 222 | 168 |
| Local cancer treatment | 3,137 | 3,355 | | 3,652 | 2,760 |
| Regional cancer treatment | 4,527 | 4,841 | | 5,269 | 3,982 |
| Distant cancer treatment | 4,527 | 4,841 | | 5,269 | 3,982 |

## Appendix Table A. Selected Model Parameters* (cont)

| **Variable** | **AFR-D,E** | **AMR-D** | **EMR-D** | **SEAR-D** | |
| --- | --- | --- | --- | --- | --- |
| Cervical cancer [15,17,25] |  |  |  |  | |
| VIA screen | 3.65 | 4.36 | 5.34 | 2.41 | |
| HPV DNA test | 8.73 | 8.88 | 9.23 | 8.48 | |
| Primary care visit †† | 2.96 | 3.04 | 2.43 | 2.76 | |
| Colposcopy + biopsy | 40 | 81 | 56 | 37 | |
| Cryotherapy | 49 | 55 | 63 | 39 | |
| Conization | 430 | 692 | 537 | 411 | |
| Local cancer treatment | 2,066 | 3,328 | 2,584 | 1,975 | |
| Regional cancer treatment | 2,238 | 3,606 | 2,799 | 2,140 | |
| Distant cancer treatment | 2,238 | 3,606 | 2,799 | 2,140 | |
| CVD [30] |  |  |  | |  |
| Blood pressure measurement ‡‡ | 0.33 | 0.33 | 0.21 | | 0.24 |
| Triple drug therapy §§ | 76 | 86 | 80 | | 76 |
| Depression [30] |  |  |  | |  |
| Questionnaire **\|\| \|\|** | 1.31 | 1.31 | 0.82 | | 0.96 |
| TCA treatment ¶¶ | 17 | 23 | 19 | | 17 |
| SSRI treatment ¶¶ | 54 | 60 | 55 | | 53 |
| Iron deficiency anemia [30,44] |  |  |  | |  |
| Hemoglobin test | 1.72 | 2.03 | 2.40 | | 1.97 |
| Iron supplement treatment *** | 3.44 | 3.52 | 2.91 | | 3.24 |
| STD [30,55,56] |  |  |  | |  |
| Syndromic management ††† | 0.65 | 0.65 | 0.41 | | 0.48 |
| PCR assay | 3.90 | 3.90 | 3.90 | | 3.90 |
| Treatment for GC and CT ‡‡‡ | 3.61 | 3.69 | 3.08 | | 3.41 |
| Treatment for GC and/or CT §§§ | 3.42 | 3.50 | 2.89 | | 3.22 |
| Treatment for PID | 127 | 127 | 127 | | 127 |

## Appendix Table A. Selected Model Parameters* (cont)

| **Test Performance** [5-7,19-23,38,43,52,53,77] **\|\| \|\| \|\|** | | Sensitivity (%) | Specificity (%) |  |
| --- | --- | --- | --- | --- |
| Clinical breast exam | | 54 | 94 |  |
| Mammogram | | 71 | 93 |  |
| VIA | | 68 | 85 |  |
| HPV DNA test | | 84 | 88 |  |
| Blood pressure measurement | | 56 | 63 |  |
| Depression questionnaire | | 98 | 75 |  |
| Hemoglobin test | | 71 | 95 |  |
| Syndromic management | | 73 | 55 |  |
| PCR assay | | 95 | 95 |  |
| * | AFR-D,E = African region; AMR-D = American region; EMR-D = Eastern Mediterranean region; SEAR-D = Southeast Asian region; D & E represent countries in those regions with highest child and adult mortality rates; HPV = human papillomavirus; CIN = cervical intraepithelial neoplasia, grade 1 (CIN 1) or grade 2,3 (CIN 2,3); CVD = cardiovascular disease; MI = myocardial infarction; STD = sexually transmitted diseases; GC = gonorrhea; CT = chlamydia; PID = pelvic inflammatory disease; VIA = visual inspection using acetic acid; DNA = deoxyribonucleic acid; TCA = tricyclic antidepressants; SSRI = selective serotonin reuptake inhibitors; PCR = polymerase chain reaction; probabilities are annual unless otherwise specified; ranges indicate age-varying probabilities. | | | |
| † | Depression transitions are 6-month probabilities. | | | |
| ‡ | STD include gonorrhea (GC) and chlamydia (CT), which can lead to pelvic inflammatory disease (PID); STD estimates are 6-month probabilities. | | | |
| § | Sequelae include chronic pelvic pain, ectopic pregnancy, and infertility; probabilities are conditional on having PID. | | | |
| **\|\|** | All-cause mortality varies with age; breast and cervical cancer mortality varies with stage of cancer. We assume no excess mortality associated with depression, iron deficiency anemia, or STD. | | | |
| ¶ | Intervention costs represent unit costs and include direct medical costs (i.e., staff, screening, lab, diagnostic, treatment, drugs); follow-up and treatment of suspected cases occur either immediately or within one year. Drug treatment for cardiovascular disease continues over 5 years. | | | |
| ** | Assumes 10 minutes of lower-level personnel time. | | | |
| †† | Cost of primary health care visit at initial screening is only included as part of the cervical cancer intervention since all other interventions, if chosen as part of the package, would occur at the same visit. | | | |
| ‡‡ | Assumes 5 minutes of lower-level personnel time. | | | |

## Appendix Table A. Selected Model Parameters* (cont)

| §§ | Includes cost of beta-blockers, statins, and aspirin and 1 primary care visit per year for 5 years; costs are discounted by 3% per year. |
| --- | --- |
| **\|\| \|\|** | Composite International Diagnostic Interview Short-Form (CIDI-SF) requires 20 minutes of lower-level personnel time. |
| ¶¶ | Includes cost of 6-month supply of antidepressant medication, 1 primary care visit, and 1 outpatient care visit over a 6-month period. |
| *** | Includes 6-month supply of iron supplements and 1 primary care visit. |
| ††† | Assumes 10 minutes of lower-level personnel time. |
| ‡‡‡ | All women who test positive with syndromic management get doxycycline (for CT) and ciprofloxacin (for GC); oral dosages are 100 mg doxycycline 2 times per day for 7 days, and 500 mg ciprofloxacin one time. |
| §§§ | Women who test positive on PCR assay get either doxycycline (22.4%), ciprofloxacin (51.1%), or both (26.5%). |
| **\|\| \|\| \|\|** | Sensitivity is defined as the probability of testing positive conditional on having disease; for VIA and HPV DNA testing, disease is defined as precancerous lesions or invasive cancer; for blood pressure measurement, positivity threshold is SBP >140 mmHG and disease is defined as CVD event; for hemoglobin test, positivity threshold is hemoglobin <119 g/L and disease is defined as anemia. |

## Appendix Table B. Average Personnel Time Required for Interventions*

| Targeted Disease | **Personnel**  **Type** | **General**  **Personnel Time**  (minutes) | Specialized Personnel Time  (minutes) |
| --- | --- | --- | --- |
| Breast cancer |  |  |  |
| Clinical breast exam (CBE) | Nurse | 10 |  |
| Mammography screen |  |  |  |
| - Preparation | Administrative | 5 |  |
| - Procedure | Technician | 20 |  |
| - Interpretation | Radiologist |  | 10 |
| Biopsy † | Surgeon |  | 20 |
| Pathology † | Pathologist |  | 10 |
| Quality control † | Pathologist |  | 5 |
| TOTAL time for CBE |  | 10 | 2 |
| TOTAL time for mammography |  | 25 | 13 |
| Cervical cancer |  |  |  |
| Preparation | Administrative | 5 |  |
| Visual inspection using acetic acid (VIA) | Nurse | 15 |  |
| HPV DNA test |  |  |  |
| - Procedure | Nurse | 10 |  |
| - Sample processing/interpretation | Pathologist |  | 10 |
| - Quality control | Pathologist |  | 5 |
| Colposcopy and biopsy † | Physician |  | 20 |
| Pathology † | Pathologist |  | 5 |
| Quality control † | Pathologist |  | 5 |
| TOTAL time for VIA |  | 20 | 6 |
| TOTAL time for HPV DNA testing |  | 15 | 21 |

## Appendix Table B. Average Personnel Time Required for Interventions* (cont)

| Targeted Disease | **Personnel**  **Type** | **General**  **Personnel Time**  (minutes) | Specialized Personnel Time  (minutes) |
| --- | --- | --- | --- |
| Cardiovascular disease |  |  |  |
| Blood pressure (BP) measurement | Administrative | 5 |  |
| Distribution of triple therapy to all women | Administrative | 2 |  |
| TOTAL time for BP intervention |  | 5 | 0 |
| TOTAL time for triple therapy intervention |  | 2 | 0 |
| Depression |  |  |  |
| Questionnaire | Nurse | 20 |  |
| TOTAL time for TCA and SSRI interventions |  | 20 | 0 |
| Iron deficiency anemia |  |  |  |
| Hemoglobin test |  |  |  |
| - Blood test | Nurse | 5 |  |
| - Sample processing/interpretation | Lab Technician | 5 |  |
| - Quality assurance | Lab Technician |  | 5 |
| Distribution of iron supplements to all | Administrative | 2 |  |
| TOTAL time for hemoglobin test intervention |  | 10 | 5 |
| TOTAL time for iron supplement intervention |  | 2 | 0 |
| STD |  |  |  |
| Syndromic management | Nurse | 10 |  |
| PCR assay test |  |  |  |
| - Sample procurement ‡ |  | 0 |  |
| - Sample processing/interpretation | Lab Technician | 10 |  |
| - Quality assurance | Lab Technician |  | 5 |
| TOTAL time for syndromic management |  | 10 | 0 |
| Total time for PCR assay test |  | 10 | 5 |

## Appendix Table B. Average Personnel Time Required for Interventions* (cont)

| * | AFR-D,E = African region; AMR-D = American region; EMR-D = Eastern Mediterranean region; SEAR-D = Southeast Asian region; D & E represent countries in those regions with highest child and adult mortality rates; HPV = human papillomavirus; DNA = deoxyribonucleic acid; TCA = tricyclic antidepressants; SSRI = selective serotonin reuptake inhibitors; STD = sexually transmitted diseases; PCR = polymerase chain reaction. Time estimates for each procedure are for one woman. These estimates were obtained from clinical experts and the published literature and reflect time required for the initial screen and for diagnostic procedures of the women who undergo further follow-up. |
| --- | --- |
| † | Only a proportion of women undergo these follow-up procedures, as calculated by the intervention models. Total time for each intervention reflects these estimated proportions of women who receive follow-up procedures. |
| ‡ | Cervical sample for PCR assay can be obtained during the cervical cancer screen. |

## Appendix Figures

**Figure A. Equations of the integer programming model applied to example.** The IP model consists of (1) the objective function, which maximizes DALYs averted through the health interventions, (2) the constraint of choosing no more than one intervention option per disease, (3) the mandatory inclusion of cervical cancer screening at age 40, (4) a budget constraint expressed as a per-woman lifetime health expenditure, and (5a,b) human resource constraints, limiting the available general and specialized personnel time separately. For each type of personnel, we calculated the time required for each disease, which was averaged between the low and high intensity intervention options. Constraints were expressed as fractions of the total amounts of general and specialized personnel times required to implement one intervention for each disease.

## Figures B-G. Schematic of disease specific models. We constructed separate state-transition models for each of the six health conditions to simulate disease incidence, prevalence, and mortality in a sequence of transitions among health states. For each region, a cohort of previously unscreened 40-year old women enters into each disease model and transitions through multiple health states until death. We assume that the health conditions are independent, but that all women are subject to background competing mortality.

## B. Breast cancer model. Health states include a healthy state and local, regional, and distant cancer states, consistent with the U.S. Surveillance, Epidemiology, End Results (SEER) classification system for cancer stages. Women face an annual risk of developing breast cancer at the local stage, which can progress to regional and distant stages over time; detection of cancer can occur through either symptoms or screening. Women can die from breast cancer or other causes.

**C. Cervical cancer model.** Health states include four categories of cervical health: healthy; infection with human papillomavirus (HPV); grade of cervical intraepithelial neoplasia (CIN); and stage of invasive cancer (i.e., local, regional, distant). Women face a monthly risk of acquiring and clearing HPV, and can progress and regress in their cervical disease. Women can die from cervical cancer or other causes.

**D. Cardiovascular disease.** Health states include a healthy state, and stroke and acute myocardial infarction (MI) states. Women face an annual risk of either stroke or MI; those who experience an event are subject to an immediate risk of death, after which they are susceptible to repeat events at higher rates.

**E. Depression.** Health states include a healthy and a depression state. Episodes of depression are assumed to be 6 months, after which recurrent episodes occur at higher rates. We assume no excess mortality associated with depression.

**F. Iron deficiency anemia.** Health states include a healthy and an anemia state. Women face an annual risk of developing anemia; once a woman develops anemia, there is no spontaneous cure and no excess mortality.

**G. Sexually transmitted diseases (excluding HIV).** Health states include a healthy and STD state, which includes two bacterial sexually transmitted infections, gonorrhea (GC) and chlamydia (CT). Women face a 6-month risk of acquiring an STD, and then progressing to acute pelvic inflammatory disease (PID), and possible sequelae (i.e., chronic pelvic pain, ectopic pregnancy, or infertility). Women with either STD or PID can have symptoms, and can experience a spontaneous cure and re-infection. There is no excess mortality associated with STD.

**Figure H.** **Discounted costs and benefits for each combination of interventions among the six diseases in AFR-D,E.** Each colored dot represents a unique package of interventions that is feasible within a specified personnel time constraint: green represents those that can be implemented when availability of both general and specialized personnel time is restricted to 50%; pink represents strategies that can be implemented if only 50% of the specialized personnel time is available; and blue represents all remaining strategies with no personnel constraints. Points A, B, and C represent the packages that are optimal when the lifetime budget is $75 per woman with no personnel constraints (A), with specialized personnel time constrained by 50% (B), and with both general and specialized personnel time constrained by 50% (C). Although the contents of the packages were different, the optimal packages under all three scenarios resulted in similar total DALYs averted. As the budget increased to $100 per woman (optimal packages shown by points D, E, and F), the impact of the personnel constraints on total DALYs averted was greater. Dotted lines represent the two budget constraints ($75 and $100 per woman).

**Figure A.**

(1)	$\text{max}\sum_{i=1}^{6} \sum_{j=1}^{2}$d*_i,j_* x*_i,j_*	where	*i* =	index for interventions:

(*i*=1 for cervical cancer; *i*=2 breast cancer; *i*=3 CVD; *i*=4 depression; *i*=5 iron deficiency anemia; *i*=6 STD)

*j* =	index for intervention	intensity:

(*j*=1 for low; *j*=2 for high)

d*_i,j_* =	DALYs averted per woman receiving intervention *i,j*

x*_i,j_* =	1 to implement programme; 0 otherwise

(2)	x*_i,1­_* + x*_i,2_* ≤ 1	for all *i*

(3)	x*_i,1­_* + x*_i,2_* = 1	for *i*=1 (cervical cancer screen)

(4)	$\sum_{i=1}^{6} \sum_{j=1}^{2}$c*_i,j_* x*_i,j_*	≤ $75 per-woman (lifetime)

where	c*_i,j_* =	average cost per woman receiving *i,j*

(5a)	$\sum_{i=1}^{6} \sum_{j=1}^{2}$g*_i,j_* x*_i,j_* ≤ p_g_ *$\sum_{i=1}^{6}$mean(g*_i,1_*, g*_i,2_*)

(5b)	$\sum_{i=1}^{6} \sum_{j=1}^{2}$s*_i,j_* x*_i,j_* ≤ p_s_ *$\sum_{i=1}^{6}$mean(s*_i,1_*, s*_i,2_*)

where g*_i,j_* =	general personnel time required for intervention *i* with intensity level *j*

s*_i,j_* =	specialized personnel time required for intervention *i* with intensity level *j*

p_g_, p_s_ =	percent between 0 and 100

**Figure B. Breast cancer model**

Healthy

Distant

Cancer

Regional

Cancer

Local

cancer

**Figure C. Cervical cancer model**


HPV

CIN 1

CIN 2,3

Distant

Cancer

Regional

Cancer

Local

cancer

Healthy

**Figure D. Cardiovascular disease (CVD) model**

Stroke

Healthy

Myocardial infarction

**Figure E. Depression model**

Healthy

Depression

**Figure F. Iron deficiency anemia model**

Healthy

Iron deficiency anemia

**Figure G. Sexually transmitted diseases (STD) model**

Healthy

STD

(GC or CT)

PID

Sequelae

**Figure H.**

DALYs averted (years)

50% shortage of available general and specialized personnel time

50% shortage of available specialized personnel time only

No shortage of available personnel time

Total Per-Woman Costs ($)

**A**

**B**

**D**

**E**

**F**

**C**
